# Heme oxygenase-1 induction in hepatocytes and non-parenchymal cells protects against liver injury during endotoxemia

**DOI:** 10.1186/1476-5926-2-S1-S42

**Published:** 2004-01-14

**Authors:** Robert B Dorman, Mary Lynn Bajt, Anwar Farhood, January Mayes, Hartmut Jaeschke

**Affiliations:** 1 Department of Pharmacology and Toxicology, University of Arkansas for Medical Sciences, Little Rock, Arkansas 72205, USA; 2 Liver Research Institute, University of Arizona, 1501 N. Campbell Avenue, Tucson, Arizona 85724, USA; 3 Department of Pathology, University of Texas Health Science Center, Houston, Texas 77030, USA

## Abstract

**Introduction:**

Heme oxygenase-1 (HO-1) is a stress response enzyme, which catalyses the breakdown of heme into biliverdin-IX alpha, carbon monoxide and ferrous iron. Under situations of oxidative stress, heat stress, ischemia/reperfusion injury or endotoxemia, HO-1 has been shown to be induced and to elicit a protective effect. The mechanism of how this protective effect is executed is unknown.

**Results:**

HO-1 induction with cobalt protoporphorin (Co-PP) dose-dependently protected against apoptotic cell death as well as neutrophil-mediated oncosis in the galactosamine/endotoxin (Gal/ET) shock model. Induction of HO-1 with Co-PP dose-dependently protected against neutrophil-mediated oncosis as indicated by attenuated ALT release and TNF-mediated apoptotic cell death as indicated by reduced caspase-3 activation. HO-1 induction did not attenuate Gal/ET-induced TNF-alpha formation. Furthermore, a similar protective effect with the high dose of Co-PP was observed when animals were treated with Gal/TNF-alpha.

**Conclusions:**

HO-1 induction attenuates apoptosis and neutrophil-mediated oncosis in the Gal/ET shock model. However, the protective effect is not due to the reduction of TNF-alpha release or the attenuation of neutrophil accumulation in the liver sinusoids.

## Introduction

Heme oxygenase (HO) catalyzes the oxidative cleavage of Fe-protoporphyrin-IX yielding equimolar amounts of biliverdin-IX alpha, free divalent iron, and carbon monoxide (CO) [1]. Among the three isoenzymes cloned to date, only heme oxygenase-1 (HO-1) can be induced by a variety of disparate stimuli, most of which are linked by their ability to provoke oxidative stress [1]. Induction of HO-1 may protect the cell against oxidative injury by a) controlling intracellular levels of "free" heme (a prooxidant), b) producing biliverdin (an antioxidant), c) improving nutritive perfusion via CO release, and d) fostering the synthesis of the Fe-binding protein ferritin [1]. In the liver, HO-1 induction protected against ischemia/reperfusion injury [2,3] and endotoxemia [4]. However, the mechanism of protection is unclear. In particular, it is controversial whether HO-1 induction in the liver protects against apoptotic and/or oncotic cell death. To address this question, we investigated the beneficial effect of HO-1 induction in the galactosamine/endotoxin (Gal/ET) shock model. Cell injury in this model involves TNF-induced apoptosis [5] as well as a neutrophil-mediated oncosis, which is caused mainly by reactive oxygen species [6,7].

## Methods

Male C3Heb/FeJ mice (Jackson Laboratories, Bar Harbor, ME) were treated i.p. with 700 mg/kg D-galactosamine (Sigma Chemical Co., St. Louis, MO) in combination with 100 micrograms/kg *Salmonella abortus equi *endotoxin (Gal/ET) or 20 micrograms/kg murine TNF-alpha (Gal/TNF). Some animals were pretreated with 5 or 15 mg/kg of cobalt-protoporphyrin (Alexis, San Diego, CA) 18 h before Gal/ET. The following parameters were measured as previously described: caspase-3 activity [5], caspase-3 processing and HO-1 expression [8], plasma ALT activities [5], plasma TNF-alpha [7], imunohistochemistry [9], neutrophil staining and histological assessment of necrosis [10].

## Results and Discussion

Western blot and immunohistochemical analysis indicated that cobalt protoporphyrin (Co-PP) dose-dependently induced HO-1 expression in hepatocytes and nonparenchymal cells, especially Kupffer cells. To investigate if increased HO-1 expression affected apoptotic or oncotic cell death, animals were treated with galactosamine/endotoxin (Gal/ET). It was previously shown that Gal/ET treatment induces TNF-alpha formation, which activates and recruits neutrophils into the liver vasculature [6] and causes a caspase-dependent parenchymal cell apoptosis [5]. In addition, the apoptotic cell death triggers neutrophil extravasation with massive aggravation of the apoptotic cell injury [5]. Gal/ET treatment caused a 10-fold increase of caspase-3 activity (indicator of apoptosis) together with substantial ALT release into the plasma (indicator of oncosis) (Table 1). Overall cell death (necrosis) was estimated to be 42 – 4%. Caspase-3 activity was reduced by 40% (5 mg/kg CoPP) or 90% (15 mg/kg Co-PP). On the other hand, ALT activities were reduced to values not different from baseline (Table 1). Neutrophil accumulation in liver sinusoids and parenchyma (174 – 10 neutrophils/50 high power fields) and adherence in venules (30 – 3 neutrophils/10 venules) were not affected by Co-PP. Furthermore, plasma TNF-alpha levels (11.6 – 1.2 ng/ml) were not reduced by Co-PP administration. These data suggest that HO-1 induction reduced both apoptotic and oncotic cell death but did not prevent production of key inflammatory mediators or neutrophil recruitment. At the low dose of Co-PP, the inhibition of neutrophil-mediated oncosis was more effective compared to attenuation of apoptosis.

**Table 1:** Hepatoprotection by Heme Oxygenase-1 Induction

	**Caspase 3 Activity (–F/min/mg protein)**	**Plasma ALT Activity (U/L)**
Controls	48 – 6	56 – 9
G/ET	479 – 23*	3700 – 900*
G/ET + 5 CoPP	289 – 28*,^#^	245 – 120*^,#^
G/ET + 15 CoPP	51 – 2^#^	51 – 14^#^
G/ET + SnPP	486 – 10*	2442 – 333*
G/ET + 5 CoPP/SnPP	573 – 94*	2486 – 679*
G/TNF	554 – 35*	1680 – 210*
G/TNF + 5 CoPP	671 – 97*	2420 – 600*
G/TNF + 15 CoPP	305 – 55*,^#^	126 – 39^#^

Apoptosis (caspase-3) and oncosis (alanine aminotransferase, ALT) were measured 7 h after Gal/ET or Gal/TNF treatment. Animals were pretreated for 18 h with cobalt protoporphyrin (5 or 15 mg/kg CoPP) and 12 h with tin protoporphyrin (15 mg/kg SnPP). Data represent means – SE of n = 5 animals per group. *P &lt; 0.05 (compared to controls) **^#^P &lt; 0.05 **(compared to Gal/ET or Gal/TNF).

To investigate if the beneficial effect was due to HO-1 induction, animals were additionally treated with the HO-1 inhibitor tin-protoporphyrin (Sn-PP). Treatment with Sn-PP alone did not affect liver injury after Gal/ET (Table 1). However, the beneficial effect of Co-PP treatment on Gal/ET-mediated apoptosis as well as neutrophil-induced oncosis was completely reversed by Sn-PP (Table 1). These data suggest that the hepatoprotection observed with Co-PP administration was mainly due to HO-1 induction.

Although the TNF response after Gal/ET could not explain the protective effect of Co-PP, the potential protection of HO-1 induction against Gal/TNF-induced liver injury was investigated. Gal/TNF caused massive caspase-3 activation (Table 1), which was confirmed by evaluation of procaspase-3 processing (data not shown). In addition, the increase in plasma ALT activities indicates oncotic cell injury. Overall, 40 – 3% of all hepatocytes were necrotic at 7 h after Gal/TNF administration. The low dose of Co-PP had no effect on any of the parameters measured. However, the high dose of Co-PP attenuated caspase-3 activities by 45% and plasma ALT activities by >90% (Table 1). The total number of necrotic cells, which were mainly from apoptotic cells, was reduced to 10 – 2%. These data suggest that the high dose of Co-PP partially reduced apoptosis but completely eliminated oncotic necrosis. In this model, hepatocellular apoptosis triggers neutrophil extravasation and cytotoxicity [5]. Elimination of apoptotic cell injury with a caspase inhibitor completely prevented neutrophil-induced liver injury [5]. In two different experiments, apoptosis was only partially prevented but oncotic cell death was almost completely eliminated by Co-PP treatment. This indicates that HO-1 induction affected both forms of cell death.

Unexpectedly, the massive HO-1 induction with increased formation of antioxidants in Kupffer cells did not attenuate TNF formation. In contrast, high doses of the antioxidant dimethyl sulfoxide eliminated TNF formation [11] and glutathione peroxidase deficiency enhanced TNF generation after Gal/ET [7]. In addition, HO-1 induction was also effective after Gal/TNF administration. Together, these data suggest that HO-1 induction in hepatocytes was more critical for the protective effect than the quantitatively higher increase of HO-1 levels in non-parenchymal cells. Moreover, an effect on hepatocellular antioxidant levels is unlikely the mechanism of protection. Recent data indicate that CO may be the more relevant mediator of the anti-apoptotic and cytoprotective effects of HO-1 induction [12,13].

## Conclusions

Induction of HO-1 in non-parenchymal cells and in hepatocytes with Co-PP dose-dependently protected against TNF-mediated apoptotic cell death and subsequent neutrophil-induced injury to hepatocytes. The protection is not due to inhibition of TNF-alpha formation or attenuation of neutrophil activation. HO-1 induction increases the resistance to both forms of cell death in hepatocytes.
